# The Behavioral Role of Digital Economy Adaptation in Sustainable Financial Literacy and Financial Inclusion

**DOI:** 10.3389/fpsyg.2021.742118

**Published:** 2021-11-11

**Authors:** Siming Liu, Leifu Gao, Khalid Latif, Ayesha Anees Dar, Muhammad Zia-UR-Rehman, Sajjad Ahmad Baig

**Affiliations:** ^1^Institute for Optimization and Decision Analytics, Liaoning Technical University, Fuxin, China; ^2^Department of Commerce, Government College University, Faisalabad, Pakistan; ^3^Faisalabad Business School, National Textile University, Faisalabad, Pakistan

**Keywords:** behavioral factors, financial literacy, financial inclusion, individual households, digital economy

## Abstract

The basic aim of this research was to investigate the impact of the behavioral biases on financial inclusion in Pakistan while considering the moderating effect of financial literacy in this relation, in the context of behavioral perspective. This study focused on the significant behavioral phenomenon, including self-control, optimism, herding, and loss aversion with a perspective of the digital economy. To test the proposed hypothesis, the primary data collection method was used. A structured questionnaire was designed to collect data from 102 individual households through the convenience sampling technique. SmartPLS was used to analyze collected data. This study found the negative impact of self-control, optimism, and herding on financial inclusion. In contrast, loss aversion contributes to the uplift of financial inclusion in Pakistan. Similarly, financial literacy proved to have a decreasing effect on financial inclusion because of religious concerns. The moderation effect of financial literacy was also significantly positive except for loss aversion. The behavioral phenomenon proved to have a significant impact on financial inclusion. This research shows that individual households who do not use developed technological services and products from formal financial inclusion can overcome the behavioral biases that hinder them from making informed financial decisions. This research work will significantly help households use financial services to improve their standard of living and overall long-term financial well-being. This research is essential because many households are not using bank services and have low financial knowledge in Pakistan. The key contribution of this research study is that it found the relation between behavioral factors and financial inclusion. Financial literacy also has a moderating effect on their relations.

## Introduction

The origin of financial inclusion is microcredit and microfinance with the emerging context of global financial exclusion. The thematic concept of financial inclusion brings access to individuals and businesses for consummating financial services and products. Financial inclusion refers to providing financial services including credit, insurance, deposits, payment, and loan services to people equally and according to their ease. It also aims to include the people in the financial circle who are either not using these services or not accessing the financial products. Although credit unions and financial intermediaries have played a significant role in providing financial assistance, almost 2.5 billion people still do not have their saving account or are not using financial services. Financial inclusion is the concept to bring all these people who are not using the financial product into the financial circle. According to major agencies that have an active role in the development of countries around the world, including the United Nations Development Programme, World Bank, G20, and Bill and Melinda Gates Foundation, there is an immense need for financial inclusion from all the sectors of the population to achieve sustainable economic growth and eliminate poverty, significantly in developing countries (Bongomin et al., 2018; Valencia et al., 2021).

According to the statistics of the State Bank of Pakistan, more than 17% (27 million) of the population of Pakistan are earning <$1 a day. Similarly, <$2 is earned by 73% or 116 million of the total population of Pakistan per day. But banks and other microfinance institutions are availed by only 2% of the poor individual. People living in rural areas are not using financial services, including account opening, bank deposits, insurance, and, most importantly, bank credit. Thus, the financial inclusion rate in Pakistan is very low. Technically, the reason behind this situation is the financial illiteracy of people about the financial concepts, so they may not access these services. Behavioral finance is comparatively a new approach in the financial area that emerged due to the problems faced by individuals in traditional finance. Behavioral finance describes the irrational behavioral phenomenon by developing the critical behavioral model. While making financial decisions, whether they are household individuals and the decision making for business, people faced different types of cognitive and emotional errors named biases that mislead them into making false decisions. Behavioral finance provides an understanding of such emotional errors and tells how people could escape from these misleading factors while making decisions among different choices. Behavioral finance emerged as a new field discussed in different newspapers, publications, and journals in the 1990s. But different authors considered that behavioral finance originated in 1800 or 1900, as was discussed in different books at that time. It is also linked with sociology, psychology, and finance (Ricciardi and Simon, 2000; Mohsin et al., 2020a).

Different types of errors connected with the cognitive and emotional abilities of an individual can affect the financial decisions due to religious, emotional, ideological, and cognitive factors; people do not behave rationally while making financial decisions among different choices (Roll, 1986; Lin et al., 2008; Sadi et al., 2011; Sarfraz et al., 2020a). These biases also affect the decisions related to financial products and services. This study considers the effects of these behavioral factors on financial inclusion. Research shows that financial inclusion helps in poverty reduction and in achieving millennium development goals. It is also found that it leads to eliminating inequality in society (Chibba, 2009; Polloni-Silva et al., 2021). Female empowerment increases as they have access to saving accounts to decide about the proper utilization of their savings. Access to credit also improves the job situation and mental health of individuals. The State bank of Pakistan has also taken the initiative to improve the statistics of financial inclusion in Pakistan, but this program is still in progress. Earnings management plays a moderating role in the Small and Medium-sized Enterprises (SMEs) cash holdings (Sarfraz et al., 2020b).

According to the Organization for Economic Co-operation and Development (OECD) working paper on finance, low financial literacy contributed to the low financial inclusion rate (Gambetta et al., 2021). Financial literacy refers to understanding the financial concepts as the individual needs to choose among different financial services. It facilitates the individuals to effectively use the financial products as it directly impacts financial inclusion (Cohen and Nelson, 2011; Naseem et al., 2020; Menyelim et al., 2021). Individuals with an understanding of the financial rules would manage their expenses efficiently, saving money leading to certain investments properly. Ignorance of financial knowledge may bring consequences to the life of an individual. By considering all this valuable information, this paper is aimed to determine the role of these behavioral biases on financial inclusion in Pakistan, considering the role of financial literacy as the moderator. Several research pieces have been conducted to evaluate the role of behavioral biases on investor behavior or the behavior of individual households while making choices among alternate financial products and services. This also helps the individual find ways to escape these mental errors and make good financial decisions. Studies have also been conducted to determine the impact of these biases on the financial behavior of an individual, how they manage their expenses and save for the future and their impact on their long-term financial well-being. But this research work is not enough in behavioral finance, and there is still scope for more contributions.

This study has the following research questions:

RQ1. What are the significant roles of behavioral errors related to the emotions and cognition of an individual while making financial decisions to use financial products or services?

RQ2. How does financial literacy affect the relationship between behavioral biases and financial inclusion?

The main objectives of this research are to determine the impact of significant behavioral biases, including self-control, optimism, herding, and loss aversion bias, on the level of financial inclusion among households in Pakistan. In addition, the role of financial literacy in relationships between behavioral biases and financial inclusion is also measured in this research masterpiece. Purposely, this study creates awareness among households to overcome their preferences in using products and services from formal financial institutions. To achieve all these objectives, we have considered behavioral biases as the independent variables, financial inclusion as the dependent variable, and the moderating role of financial literacy.

The intangibility concept of low financial inclusion with a behavioral approach is the core phenomenon of intellectual capital. First, this thematic research is argued for current financial sector services and competitive advantages in this digital era. Second, the focused country of this research is Pakistan which is not even discussed in the existing literature. The focused country is essential in the broad sense that Pakistan has a mixture of banking and financial inclusion services (Islamic and Conventional) and a developing economy. These two elements, i.e., Islamic state and developing economy, declare Pakistan an overheated economy that enhances the exciting aspect of being a focused research country. Third, the Smart-PLS is used to empirically examine the impact of behavioral causes of low financial inclusion in an Islamic and developing country. Lastly, this research is made some recommendations for enhancing the competitive advantages for Pakistan in the sustainable development of financial inclusion.

## Literature Review

### Financial Inclusion

Financial inclusion refers to “providing the financial services and products to the poor and disadvantaged people on affordable cost and equivalent basis” (Dev, 2006). Financial inclusion aims to involve all those individuals into the monetary circle who are either unbanked or underbanked. Thus, the individuals who do not have a bank account or do not have access to financial services are all financially excluded. There is a figure of the world population which has omitted financially. According to the United Nations survey, nearly three billion people worldwide have not accessed the financial services and products offered by banks or other financial institutions such as a safe place to save money, credit, loan, and insurance. Mostly the financially excluded people in developing countries are the average class people. Providing financial services to individuals to handle money in formal ways will help them reduce poverty and help achieve millennium development goals (Chibba, 2009; Mohsin et al., 2020b). Countries like Pakistan are resource-constrained and face additional challenges for financial inclusion than a developed country. Globally, there are gender differences that are more severe in developing countries in handling financial recourses, security, and empowerment (Ibtasam et al., 2018).

Advancement and expansion of the banking system in Pakistan due to privatization and solid financial policies have shown a significant deepening of the inclusive financial system. But the financial system is not fully expanded to all the sectors of society, especially in rural areas. In Pakistan, financial exclusion is caused by many reasons, including geographical locations as 67% of people live in rural areas, cultural or language barriers, banking services, behavior, lack of support at provincial levels, regulatory restraints including money laundering, and suitability of financial products (Menyelim et al., 2021). The efficient policies of state banks and initiatives by the government have not yet achieved the goals of including people into the financial circle completely. According to Husain (2011), one of the fundamental problems in expanding final inclusion in Pakistan is mismatching the long-term loans and short-term deposits of bank customers. But this problem is resolved, and the gap is filled to some extent by the long-term Islamic Sukuk bonds, insurance by different institutions, pension funds, municipal bonds, and endowment funds. But the progress in financial inclusion is plodding than the developed nations.

Financial inclusion has become a research and policy concern in many countries around the world. State banks worldwide, along with World Bank, have taken the initiative to improve financial inclusion statistics. Still, there are a variety of factors to be investigated that cause low inclusion in financial systems. The innovativeness of this research idea lies in its concept, as no such research study has been conducted. The behavioral causes of low financial inclusion in Pakistan are still unknown in research fields. So, this could be assumed that current research will not only contribute to making people aware of taking control of their business to include into the financial system but also help to achieve the financial inclusion goals of the state bank of Pakistan. It will also contribute to strengthening the banking system in Pakistan.

#### Behavioral Bias

The word bias refers to the tendency of an individual toward a conclusion or disposition. Biasness is a particular type of tool or specific design used by the human mind to handle the overloaded information and conclude decision-making. As these biases impact behavior or choices in the decision-making process, they need to be further researched to gain more insight into these concepts (Sahi and Arora, 2012). Behavioral finance literature considers biases as deviations from certain norms: cognitive limitations, heuristic, or information processing strategies (Tversky and Kahneman, 1974). Researchers in the psychological area observed that people sometimes show abnormal behavior while making decisions. The main reason behind poor decision-making may be cognitive errors or emotional imbalances (Sarfraz et al., 2018; Baig et al., 2019).

#### Self-Control Bias

Self-control bias is defined by Pompian (2011) as the tendency of the individuals that causes them to consume today at the expense of tomorrow. Self-control is considered the conflict between the overarching desires of individuals and their inability to act according to these desires due to the lack of self-discipline. Self-control is interchangeable with self-discipline as it is the ability to put down some solid responses for achieving some higher goals. Still, these restrictions are not automatic and require conscious efforts (Duckworth and Seligman, 2006). Individuals with low self-control tend to be more self-centered and impulsive, enjoy ease, and become riskier. In addition, people with low self-control have less ability to calculate the consequences of their bad financial decisions (Wolfe and Higgins, 2009).

#### Self-Control Bias and Financial Inclusion

Research findings proved the positive impact of self-control on the financial behavior and financial well-being of individuals. People with better self-control are more likely to save money from every paycheck, have satisfaction through their financial behavior, and are less likely to be anxious and feel secure in their current and future financial matters (Strömbäck et al., 2017).

#### Behavioral Life-Cycle Hypothesis

According to Pompian (2011), the behavioral life-cycle hypothesis provides the best technical description of self-control in saving and consumption. The individual saving decisions represent their preference for present over future consumption. Previous literature shows that propensity of individuals to save, budget, and make better financial decisions largely depends on their financial behavior control. Similarly, the individuals who are future-oriented and have financial knowledge about the financial terms and rules are more likely to save for their future and participate in different retirement plans, and this will result in controlling their consumptions today (Perry and Morris, 2005; Howlett et al., 2008).

Studies have also investigated the link between self-control and financial behavior while making choices among financial products and services, for example, bank credit and retirement plans. Households lacking commitment, monitoring, and financial planning also have accumulated less wealth (Strömbäck et al., 2017). Thus, the individual with self-discipline is more likely to save for the future and get the financial services to accumulate the money or get a place to save the money. This study aims to explore the relation between self-control and financial inclusion. It argues that people with high self-discipline in their monetary and saving behavior are more likely to use banks and financial services and products of different financial institutions. On the contrary, individuals with accumulated wealth are more likely to use bank accounts to deposit money, bank credit, insurance, retirement plans, and investment in different funds. Furthermore, self-control contributes to increasing financial inclusion and accumulating people in the financial circle.

***H***_**1**_***: There is a significant relationship between self-control and financial inclusion***.

#### Optimism Bias

A valuable definition of optimism was given by Peterson (2000): “an attitude or mood about the material or future social expectations, on which the person having the expectations are socially desirable, either because of his pleasure or his advantage.” There is not a single optimism; it depends upon the contents because what is desirable for a person will be his optimism. Individuals are more optimistic when they can control the outcomes or have a high commitment (Heaton, 2002).

#### Optimism Bias and Financial Inclusion

When predicting what will happen with us in the future, next year or 50 years from now, we underestimate the probability of adverse events and overestimate the probability of positive events. We expect to live long, overestimate our professional success, and believe that our children will be talented. This phenomenon is known as optimism bias and is one of the most prevalent behavioral finance concepts and economics (Sharot et al., 2012; Naseem et al., 2021). One of the main objectives of this study is to investigate the relationship between optimism and financial inclusion. As optimistic persons see their future with “rose-colored glasses,” they only expect good things to happen in the future. Thus, people with an upbeat nature are more likely to think that their future will be risk-free. Such people say they will remain financially strong in the future and do not need to save for rainy days, which results in not using the services of financial institutions. As a result, they may be less willing to invest their savings into different financial products, including insurance, fund investment.

***H***_**2**_***: There is a significant relationship between optimism and financial inclusion***.

#### Herding Bias

Herding is the behavioral phenomenon that primarily originated from the animals moving in groups and following one another has been widely studied in financial markets, especially in stock markets. Zhang and Chen (2017) define herding by considering it as: “individuals doing what other individuals are doing, even when their information suggests them to do something different from the others.” Literature has classified herding behavior into two major types, whether herding is rational or irrational. Irrational herding could be observed when individuals follow the choices of others because they consider it part of social norms. Irrational herding occurs due to learning through the observations of an individual (Zhang and Liu, 2012).

#### Herding Bias and Financial Inclusion

Herding is often observed when people follow the same choices, and most of them do this to mimic the actions of others. Herding behavior based on experiments conducted includes different economic activities, such as earnings forecasts, investment recommendation, corporate conservatism, and initial public offerings (Graham, 1999). People mostly get influenced by the choices of others in their purchase decisions. Although herding has often received negative comments, the literature reports the positive impact of the recommendations of online consumers on other consumer choices (Huang and Chen, 2006). In the light of these entire viewpoints, it is argued that household individuals exhibit herding behavior when choosing among alternatives in their financial decisions. Pakistani people, mostly, the rural areas and the poor who do not have financial knowledge, do not consider bank credit as the source when they need money. They mostly get loans from their relatives and friends instead of considering banks as an opportunity. Thus, this study aimed to investigate the relation between the herding behavior of individual households and the probability of their financial inclusion by considering all these literature viewpoints.

***H***_**3**_***: There is a significant relationship between herding and financial inclusion***.

#### Loss Aversion Bias

Loss aversion has been described as a similar loss or gain with different mental penalty levels for an individual. The research evidence shows that people exhibit more distress when they face a loss than pleasure from equal gain. Similarly, the loss coming after the prior loss is more painful in a usual situation than the loss after the earning. Thus, avoiding the risk is common in households that can affect their household financial decisions (Ngoc, 2014). Simultaneously, making forecasting about how outcomes of decisions feel, people believe that the hedonic impact of loss will be superior to the hedonic impression of equal sizes gain. If the prediction asymmetry is identical to the actual results, people will be wise in their decisions (Kermer et al., 2006).

Loss aversion is based on the researches of two psychologists Daniel Kahneman and Amos Tversky in 1979. Khan (2017) found that people are satisfied from gain but double ache when they have losses. They refer to several studies based on cultural effects on the loss aversion nature of the individual. According to them in Pakistan, women are more loss averse than men in financial decision-making. Similarly, unemployed and older adults near retirement are more loss averse than young and employed individuals.

#### Loss Aversion Bias and Financial Inclusion

Originally, loss aversion bias was studied in gamblers for their two-outcome monetary choices. But within a short time, researchers have made significant studies in different fields like marketing, consumer choice, and psychology.

#### Prospect Theory

Formally, the loss aversion could be described based on prospect theory developed by Daniel Kahneman and Amos Tversky in 1979 and 1992, the widely used and known theory for deciding risk. According to the prospect theory, people judge the outcomes of their decisions in terms of gain or losses, concerning some reference point, and they have more sensitivity for losses than the gains. The drawback of loss aversion is people have more probability weightage for the losses than the probability estimation for gains (Abdellaoui et al., 2007). Prospect theory defines human behavior when they are under the situation of risk and uncertainty. It is also linked with status quo bias. The reference point of the outcomes is also considered the status quo, against which any loss is not bearable for the people (Köbberling and Wakker, 2005).

Prospect theory relates the loss aversion to the consumption/saving behavior of the individuals. Fisher and Montalto (2011) conducted a research study using the U.S. survey of consumer financial data to determine the saving behavior of households. The research findings confirmed the asymmetric impact of good or bad news related to the income of an individual on his/her saving behavior. Households are loss averse, as the increase in the income above the reference level does not significantly relate to savings chances. In light of all these research contributions, this study aimed to investigate the role of loss aversion on the financial inclusion of an individual household. Loss-averse people have fewer intentions toward saving even when their income is above the reference level. Similarly, the loss-averse behavior of individuals could also affect the choices of financial services provided by banks and other financial institutions.

***H***_**4**_***: There is a significant relationship between loss aversion and financial inclusion***.

### Financial Literacy

Different researchers and organizations have defined financial literacy. The President's Advisory Council on Financial Literacy (PACFL) provided the most common definition in 2008: “the management of financial resources for people's lifetime well-being by using knowledge and skills.” In literature, financial literacy is defined in many ways as good financial behavior, financial knowledge, perceived knowledge, skills, and abilities to apply financial knowledge and financial experiences. According to Lusardi and Mitchell (2008), financial literacy is “understanding about the basic concepts of finance, such as the real and nominal values differences, diversification of basic risks and the phenomenon of interest compounding.”

#### Financial Literacy and Financial Inclusion

There are different views regarding the impact of financial literacy of individuals on financial behavior. Literature provides evidence that the impact of financial literacy on the overall financial behavior is not too certain, although it is observed that financial behavior is positively influenced by financial literacy. Bernheim et al. (2001) investigated the relation of financial literacy with saving behavior. Those who studied the finance course in their high school were observed to have more savings in their middle age than those who did not study the finance subjects.

The long-term saving behavior of households is also linked to financial literacy. Research studies have investigated the role of financial literacy in planning financial decisions among older women. Evidence proved the interrelation between financial literacy and planning; women with financial knowledge are expected to make better financial decisions and long-term planning to save for retirement (Lusardi and Mitchell, 2008; Naiwen et al., 2021). Financial literacy has been studied about various behavioral aspects, including long-term well-being, investment decision-making behavior, and financial decision-making of individuals. Research evidence also provided information about the positive role of financial literacy in enhancing financial inclusion in different countries. The more people know about financial terms, the more they can make financial decisions and escape behavioral biases. The present research considers the moderating role of financial literacy in the relation between behavioral biases and financial inclusion.

***H***_**5**_***: Financial literacy moderates the relationship between self-control and financial inclusion***.
***H***_**6**_***: Financial literacy moderates the relationship between optimism and financial inclusion***.
***H***_**7**_***: Financial literacy moderates the relationship between herding and financial inclusion***.
***H***_**8**_***: Financial literacy moderates the relationship between loss aversion and financial inclusion***.

### Theoretical Framework

**Figure 1:**
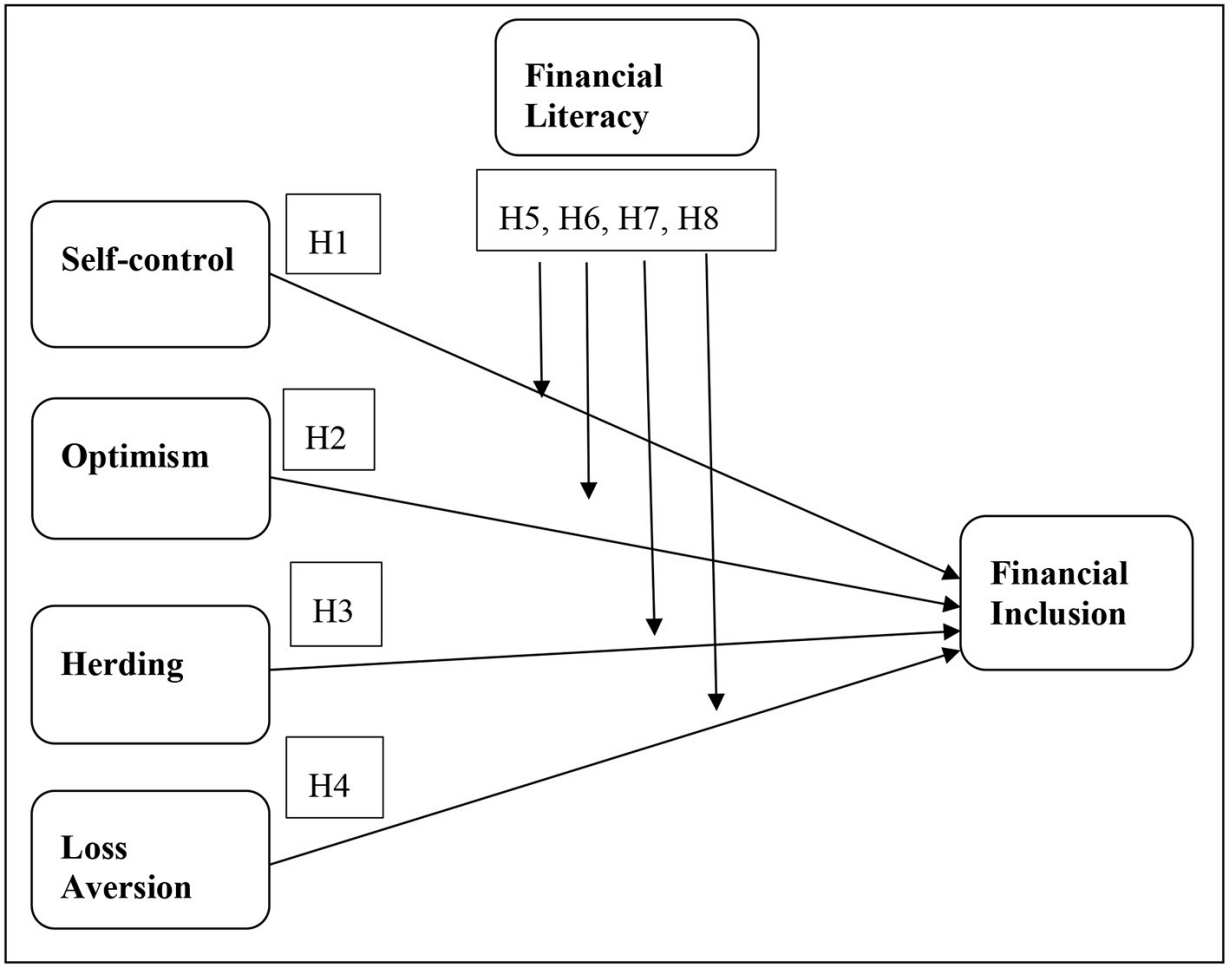
Theoretical model.

In light of the detailed information provided in previous literature, I have developed the theoretical model. This research work aims to investigate the impact of behavioral biases on the financial inclusion of a household individual. This research study considered self-control, optimism, herding, and loss aversion as behavioral biases. Although there are more than 100 biases in the behavioral finance literature, I have worked on only four. Financial inclusion is considered as a dependent variable, while financial literacy is the moderating variable. The detailed review of the previous literature provided the basis for the arguments that behavioral biases are linked to the long-term financial decision-making of an individual. The overall financial well-being depends on the information a person has about financial products and services (Figure 1).

## Data Description and Methodology

### Data Description

This research study aims to determine whether behavioral biases have a role in the financial inclusion of a household individual when choosing the products and services in financial markets. Thus, all the household individuals of Pakistan are considered as the population for the study. The individuals included in the study belong to various professions, including banking, textile, teaching, shopkeepers, etc. This research also consists of people from both the private and public sectors. The population for this research study is a huge number, so the sample is derived, which is considered the subset of the representative of the whole population. As the number of household individuals using the financial products and services is too large, we could not collect data from each one of them, so it took 102 respondents as a sample.

This research study is based on common household individuals. It does not require any specific professional knowledge or skills, which allowed us to use the convenience sampling technique to collect data from the respondents. Data are collected from every possible individual willing to respond, but the respondent must have some post qualification job experience. This condition was applied with the thought that individuals working must have some savings, which will genuinely measure their self-control nature. It will also help understand the behavioral errors that restrict their decision-making ability to use financial market services, especially banks. Research data are analyzed with the use of software including SPSS and smartPLS.

### Questionnaire

The questionnaire for this research consisted of two main parts: demographics and variables. The demographic portion of the questionnaire consists of variables including gender, age, community, income, occupation, and marital status. The second part of the questionnaire consists of the items of different variables. The independent variable self-control consists of 4 items, optimism 5 items, herding 4 items, and loss aversion 3 items. The dependent variable is financial inclusion having 7 items measure it. Financial literacy has been considered as the moderator, which includes 5 items.

### Measures

This quantitative study has adopted measures for the collection of data. Self-control consists of 4 items adopted from Antonides et al. (2011); 5 items for optimism have been derived from Scheier and Carver (1985); 4 items for variable herding have been adopted from Metawa et al. (2018); 3 items for the loss aversion have been derived from two different sources (Ngoc, 2014; Baker, 2018). Financial inclusion is measured by 7 items that have been derived from Demirgüç-Kunt et al. (2015). All these variables have been measured on five-point agreement Likert scale. The values ranged from disagreement to agreement where 1 = strongly disagree, 2 = disagree, 3 = neutral, 4 = agree, 5 = strongly agree.

Financial literacy consisted of 5 items to measure the financial knowledge of the individual. All these items are adopted from Rooij et al. (2011) and Menyelim et al. (2021). The nominal scale measures these items (Table 1).

**Table 1:** Previous research glimpses.

**Sr. No**.	**Variables**	**Questions adopted from**	**No. of items**
1	Self-control	Antonides et al., 2011; Strömbäck et al., 2017	4
2	Optimism	Scheier and Carver, 1985; Strömbäck et al., 2017	5
3	Herding	Metawa et al., 2018	4
4	Loss aversion	Ngoc, 2014; Baker, 2018	3
5	Financial inclusion	Demirgüç-Kunt et al., 2015	7
6	Financial literacy	Rooij et al. (2011)	5

## Results and Discussion

### Model Characteristics

The research study aimed to determine the relationship between behavioral biases and financial inclusion in Pakistan while considering financial literacy as a moderator. Thus, the questionnaire was distributed among the individual to collect data. Data analysis software, including SPSS and Smart PLS, was used for data analysis.

### Evaluation of Measurement Model

The measurement model for this research study is evaluated based on reliability and validity. The most important measures of reliability are composite reliability and factor loadings. The reliability at the item and construct level is satisfied if it exceeds the threshold level of 0.50 and 0.70 (Zia-ur-rehman et al., 2017; Gambetta et al., 2021). The composite reliability and the factor loading of the constructs are shown in Table 2.

**Table 2:** Construct reliability and validity.

	**Cronbach's alpha**	**CR**	**AVE**
Financial inclusion	0.743	0.822	0.540
Financial literacy	0.693	0.785	0.582
Herding	0.716	0.667	0.504
Herding × financial literacy	0.650	0.636	0.519
Loss aversion	0.711	0.783	0.580
Loss aversion × financial literacy	0.655	0.660	0.565
Optimism	0.888	0.796	0.661
Optimism × financial literacy	0.726	0.655	0.574
Self-control	0.624	0.730	0.605
Self-control × financial literacy	0.779	0.624	0.584

*CR, composite reliability; AVE, average variance extracted*.

### Discriminant Validity

Discriminant validity is measured using the Fornell and Larcker (1981) test, which describes that the square root should be greater than the correlation between other constructs in the rows and columns. Thus, discriminant validity is the criteria that measure the difference of a variable from the others (Zia-ur-rehman et al., 2017; Gambetta et al., 2021). Table 3 represents the validity of the variables in the research study.

**Table 3:** Discriminant validity.

	**Financial inclusion**	**Financial literacy**	**Herding**	**Loss aversion**	**Optimism**	**Self-control**
Financial inclusion	0.663					
Financial literacy	−0.295	0.697				
Herding	−0.230	0.137	0.710			
Loss aversion	0.238	−0.171	0.182	0.808		
Optimism	−0.128	0.037	0.312	0.098	0.813	
Self-control	−0.244	0.006	0.373	0.101	0.309	0.777

### Assessment of Structural Model

The predictive capacity of the model has been determined by the significance of path coefficients and the determination coefficients. To obtain the significance level and the path coefficient values, bootstrapping was run at 300 points. The resulting values obtained have been presented Table 3.

### Coefficient of Determination

The coefficient of determination criteria is that the acceptable *R* square having values 0.75, 0.50, and 0.25 describes the relatively substantial, moderate, and weak coefficient of determination (Hair et al., 2012; Gálvez-Sánchez et al., 2021). As the *R* square value of the financial inclusion presented in Table 4 is 0.418, it represents moderate predictive power.

**Table 4:** *R* square.

	***R* square**	**Predictive accuracy**
Financial inclusion	0.418	Moderate

Another important criterion to access the structural model is the measurement of square value. According to the effect size of the constructs omitted on the endogenous constructs, it is determined as small, medium, and large based on relative values 0.02, 0.15, and 0.35, respectively. Table 5 represents the *f* square value of the variables in different relations (Gambetta et al., 2021).

**Table 5:** *f* square.

	***f* square**	**Effect size**
Financial inclusion		
Financial literacy	0.083	Medium
Herding	0.031	Medium
Herding × financial literacy	0.156	Medium
Loss aversion	0.076	Medium
Loss aversion × financial literacy	0.036	Medium
Optimism	0.000	Small
Optimism × financial literacy	0.000	Small
Self-control	0.036	Medium
Self-control × financial literacy	0.025	Medium

**Figure 2:**
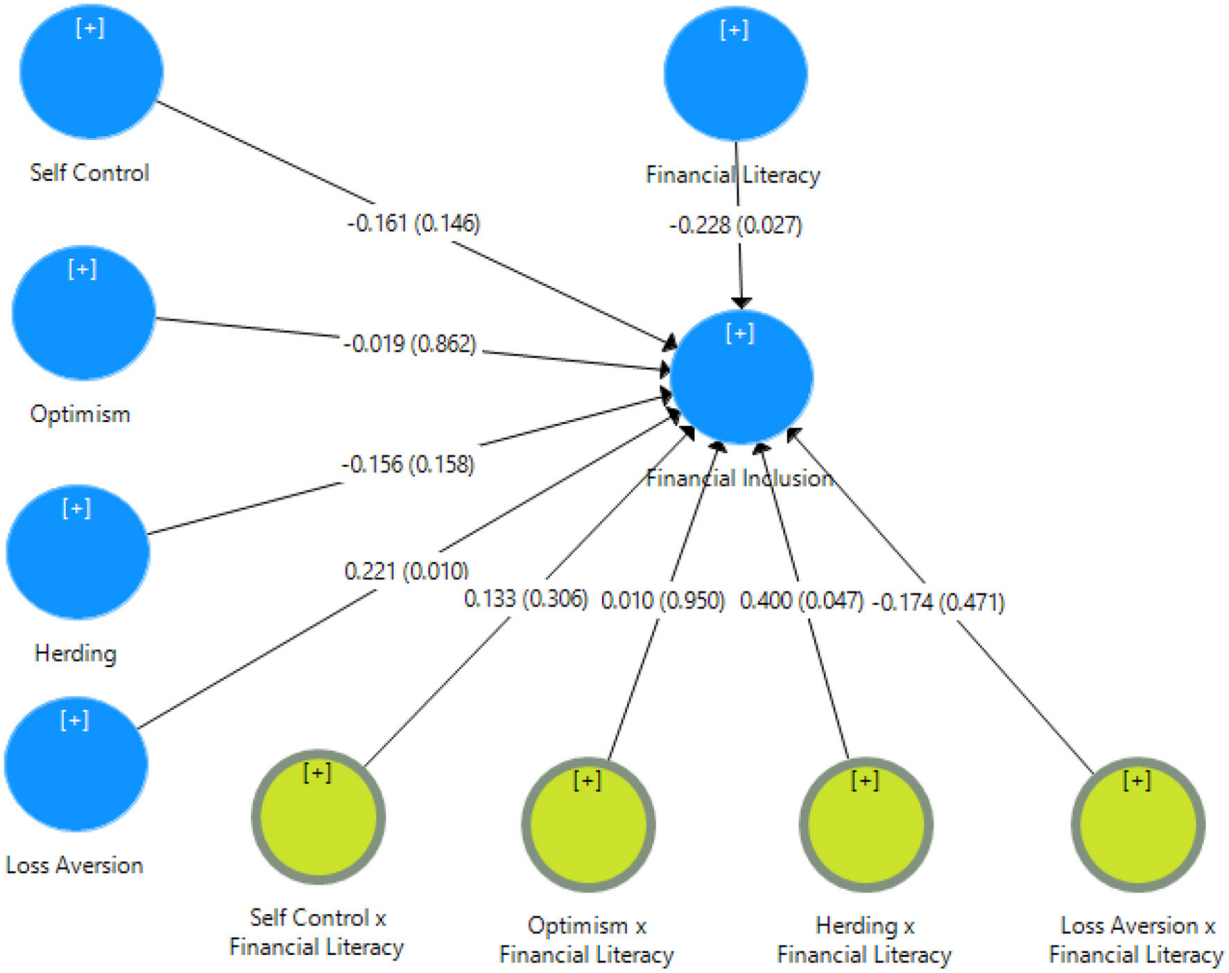
Path coefficients in PLS-SEM model.

The last part of the analysis consists of the determination of the path coefficient. The path coefficient determines whether the collected data support the hypothesis or not. The +1 value of the coefficient shows strong positive relation while the −1 shows strong negative relations. Table 6 represents the value of the path coefficient for each relation, along with the *p*-value. A *p* < 0.10 is significant to support the hypothesis (Figure 2).

**Table 6:** Path coefficients.

**Hypothesis**	**Relationship**	**Path coefficients**	***p*-value**	**Decision**
H1	Financial literacy -> Financial inclusion	−0.228	0.027	Supported
H2	Herding -> Financial inclusion	−0.156	0.158	Not supported
H3	Herding x Financial literacy -> Financial inclusion	0.400	0.047	Supported
H4	Loss aversion -> Financial inclusion	0.221	0.010	Supported
H5	Loss aversion x Financial literacy -> Financial inclusion	−0.174	0.471	Not supported
H6	Optimism -> Financial inclusion	−0.019	0.862	Not supported
H7	Optimism x Financial literacy -> Financial inclusion	0.010	0.950	Not supported
H8	Self-control -> Financial inclusion	−0.161	0.146	Not supported
H9	Self-control x Financial literacy -> Financial inclusion	0.133	0.306	Not supported

## Discussion

The study aimed to explore all the behavior factors that caused low financial inclusion in Pakistan. A small proportion of the total population in Pakistan uses financial products and services. To analyze the results, we use SPSS and smartPLS. The results identified whether the findings had supported the hypothesis we developed or not. The H_1_ hypothesis argues that there is a significant relationship between self-control and financial inclusion. Still, results suggest an insignificant negative relationship between self-control and financial inclusion with values (β = −0.161, ρ = 0.146). However, the researchers argue the financial satisfaction and habitual saving of money among the individuals having self-control in their financial matters (Strömbäck et al., 2017). That will be expected to increase their usage of financial services to deposit money. The negative relation between self-control and financial inclusion indicates that people may have savings but not use banking services because of cultural barriers and religious beliefs. Especially in rural areas, Pakistani people prefer to deposit money with their relatives because they lack knowledge or access to financial services. The second hypothesis elaborates as the relation between optimism and financial inclusion is insignificantly negative (β = −0.019, ρ = 0.862), as the highly optimistic people do not consider saving for the future or investing in opportunities. As Peterson (2000) described, bright people judge their future on their expectations based on pleasure or desirable outcomes; they overestimate the likelihood of positive events and underestimate the likelihood of negative events (Sharot et al., 2012). The findings of the current research study are also aligned with previous literature as the people with “rose eyed glasses” may think their future is financially safe, so they do not need to plan for future financial events. This will reduce their savings and also the usage of financial products and services. The third hypothesis is justified by analytical process which confirmed that there is also a negative relationship between herding and financial inclusion (β = −0.156, ρ = 0.158) because herding involves irrational decision-making without the informed knowledge.

Zhang and Chen (2017) argue that people show herding behavior when following choices of others without rational decisions. For example, most people in Pakistan make their financial decisions by observing the behavior of others because of a lack of financial knowledge. There is also the possibility that they will not consider banking and other financial institutions as opportunities to avail of financial services due to their herding behavior, so these findings align with the arguments of previous researchers. The loss of aversion and financial inclusion are significantly positively related (β = 221, ρ = 0.010). People are most sensitive to losses, as they feel more distress from a loss than pleasure from an equal amount of gain (Ngoc, 2014; Gálvez-Sánchez et al., 2021). This loss aversion behavior is expected in individuals to place their money with secure investments. Banks and other financial institutions are safe and secure options for depositing money and investing; it is expected to increase financial inclusion due to the loss aversion nature of individuals. There is a significantly negative relationship between financial inclusion and financial literacy (β = −0.228, ρ = 0.027), although previous research found a positive impact of financial literacy on financial inclusion.

For example, Bernheim et al. (2001) argued that individuals who studied finance courses are more likely to save in the future and use financial services. In Pakistan, this negative relation exhibits low participation of people in banking services because of their religious beliefs. The majority of the population of Pakistan is Muslim; people avoid the conventional banking system because of the interest element prohibited in Islam. Thus, they may have the literacy of financial matters, but their religious and cultural factors forced them to avoid banking services. Similarly, the research findings demonstrate the positive impact of moderating variable financial literacy on the relationship between independent variables self-control, optimism, and herding with dependent variable financial literacy by values (β = 0.133, ρ = 0.306), (β = 0.010, ρ = 0.950), and (β = 0.400, ρ = 0.047), respectively. Thus, the moderator contributes to the lowering strength of the relationship between loss aversion and financial inclusion.

## Conclusion

Without knowing the emotional and cognitive domains of behavior, it will be difficult for an individual to make the right decision among the prevailing financial choices. This research indicates that individual behavior is biased when they decide to take financial services. Individual decision-making behavioral biases strongly cause the low rate of financial inclusion in Pakistan. The fundamental aim of this research was to investigate the moderating role of financial literacy and how the literacy level of an individual could play a role in overcoming these biases and increasing financial inclusion. The analysis of the data collected by 102 respondents proved the negative impact of self-control, optimism, and herding biases on financial inclusion. Individuals may have savings, but they do not consider the potential investment opportunities of banks and other financial institutions. Their religious beliefs are a major cause of this issue with the banking system.

The optimistic nature of individuals also contributes to the low inclusion in Pakistan. People who are highly encouraging see their future positively and do not invest in potential financial products. Optimism is also proved to have a negative relation with financial inclusion. Furthermore, herding behavior results in low inclusion in Pakistan. An individual may herd the irrational choices of other people that are also a leading factor of low inclusion. Only loss aversion positively impacts financial inclusion because the individuals who know the banking system and its terms do not consider it a loss and avail of financial services. Research findings suggest that financial literacy as a moderator positively impacts the relationship between biases and financial inclusion, except for the relationship between loss aversion and financial inclusion. Therefore, the increasing rate of literacy could improve the inclusion level in Pakistan. The negative relation between financial literacy and financial inclusion indicates the low participation of the individual in banking services because of their religious beliefs. In Pakistan, Muslims have strict behavior toward interest, as interest is prohibited in Islam. So, the religious beliefs of the individual restrict them from using banking services. Based on the findings of this research work, a framework could be designed to create awareness and enhance the financial literacy level of households, which will help them recognize financial opportunities by banks and other institutions. Banks also have the advantage of this study as it will help increase their customer base by overcoming the biases of household individuals toward financial decisions. This will also contribute to the financial inclusion program of the State Bank of Pakistan, as increasing individuals into the financial circle will contribute to achieving its financial inclusion goals.

### Limitations and Future Directions

The sample size for the study was significantly less because of the short time. Further researches can increase the number of respondents. Future researchers could also consider other biases (mental accounting, availability, conservatism) that may have also caused the low financial inclusion of Pakistan. The research studies could consider the cross-cultural aspects of the population and compare the financial inclusion in rural and urban areas.

## Data Availability Statement

The raw data supporting the conclusions of this article will be made available by the authors, without undue reservation.

## Author Contributions

SL and LG: conceptualization. AD and MZ-U-R: methodology. SB and SL: formal analysis and investigation. AD: writing—original draft preparation. SL: writing—review and editing. LG: resources. KL and LG: supervision. All authors approved the current study.

## Conflict of Interest

The authors declare that the research was conducted in the absence of any commercial or financial relationships that could be construed as a potential conflict of interest.

## Publisher's Note

All claims expressed in this article are solely those of the authors and do not necessarily represent those of their affiliated organizations, or those of the publisher, the editors and the reviewers. Any product that may be evaluated in this article, or claim that may be made by its manufacturer, is not guaranteed or endorsed by the publisher.
